# Gold(III) bisdithiolate complexes: molecular conductors that also exhibit anticancer and antimicrobial activities

**DOI:** 10.1080/07853890.2021.1896913

**Published:** 2021-09-28

**Authors:** Fernanda Marques, Sílvia A. Sousa, Jorge H. Leitão, Tânia S. Morais, Yann Le Gal, Dominique Lorcy

**Affiliations:** a Centro de Ciências e Tecnologias Nucleares, Instituto Superior Técnico, Universidade de Lisboa, Bobadela, Portugal; b iBB-Institute for Bioengineering and Biosciences, Departmento de Bioengenharia, Instituto Superior Técnico, Universidade de Lisboa, Lisboa, Portugal; c Centro de Química Estrutural, Faculdade de Ciências, Universidade de Lisboa, Lisboa, Portugal; d Departamento de Química e Bioquímica, Faculdade de Ciências, Universidade de Lisboa, Lisboa, Portugal; e Institut des Sciences Chimiques de Rennes UMR 6226, Université de Rennes 1, Rennes, France

## Abstract

**Introduction:**

Au(III) complexes with dithiolene ligands have been used as molecular conductors and magnetic materials [1]. These compounds feature square planar geometries, as that for cisplatin the Pt(II)-based compound in clinical use as chemotherapeutic drug. Based on the molecular structure, a similar mechanism of action, e.g. antitumor activity and interaction with DNA should be expected for these compounds. Reports on the biological activity of similar gold(III) compounds are relatively scarce in the literature. Only recently, a Au(III) 1,2-dithiolene cyclometalated complex had proved its potential against Gram-positive bacteria [2]. In this study we evaluated two related Au(III) complexes containing N-alkyl-1,3-thiazoline-2-thione dithiolate ligand, [Au(R-thiazdt)_2_]^−1^ (R = ethyl-**1**; R = hydroxyethyl-**2**) [3a,b] as antitumor and antimicrobial agents. The compounds were assessed *in vitro* towards cisplatin sensitive ovarian cancer cells (A2780), bacteria and fungus of clinical importance such as *Staphylococcus aureus* and *Candida*. Spectroscopic studies were also performed to evaluate the interaction with DNA.

**Materials and methods:**

The gold complexes were synthesised as previously described [3a,b]. The cytotoxic activity against the A2780 ovarian cancer cells was assessed by the IC_50_ determined by the MTT assay. The antimicrobial activities of complexes **1** and **2** were assessed by the MIC values towards the Gram+ *S. aureus*, and the fungal strains *C. glabrata* and *C. albicans*, using reported methods [4,5]. The ability of compounds to bind to DNA was assessed by fluorescence spectroscopy using ethidium bromide (EB) as the fluorescence probe.

**Results:**

Complexes **1** and **2** presented high cytotoxic activity in the cisplatin sensitive A2780 cells even superior than cisplatin. Complex **1** was able to inhibit the growth of *S. aureus* and both *Candida* strains, while **2** was much less active. The fluorescence studies revealed a weak interaction of compounds with CT-DNA, in contrast with that found for cisplatin, although the interaction of **2** is somewhat stronger than that found for **1**.

**Discussion and conclusions:**

Results evidenced the importance in what way minor modifications of the Au dithiolate structure can result in loss of activity in particular the antimicrobial activity. In contrast with cisplatin, DNA is not the main target involved in their mode of action. Further studies are needed to explore other potential targets and the mechanism of action.
